# Supplementary figures and images for: First detection of Crimean Congo Hemorrhagic Fever antibodies in cattle and wildlife of southern continental France: Investigation of explanatory factors
Source: PLoS One. 2025 Sep 24;20(9):e0331875. doi: 10.1371/journal.pone.0331875 (PMC12459774; doi:10.1371/journal.pone.0331875)

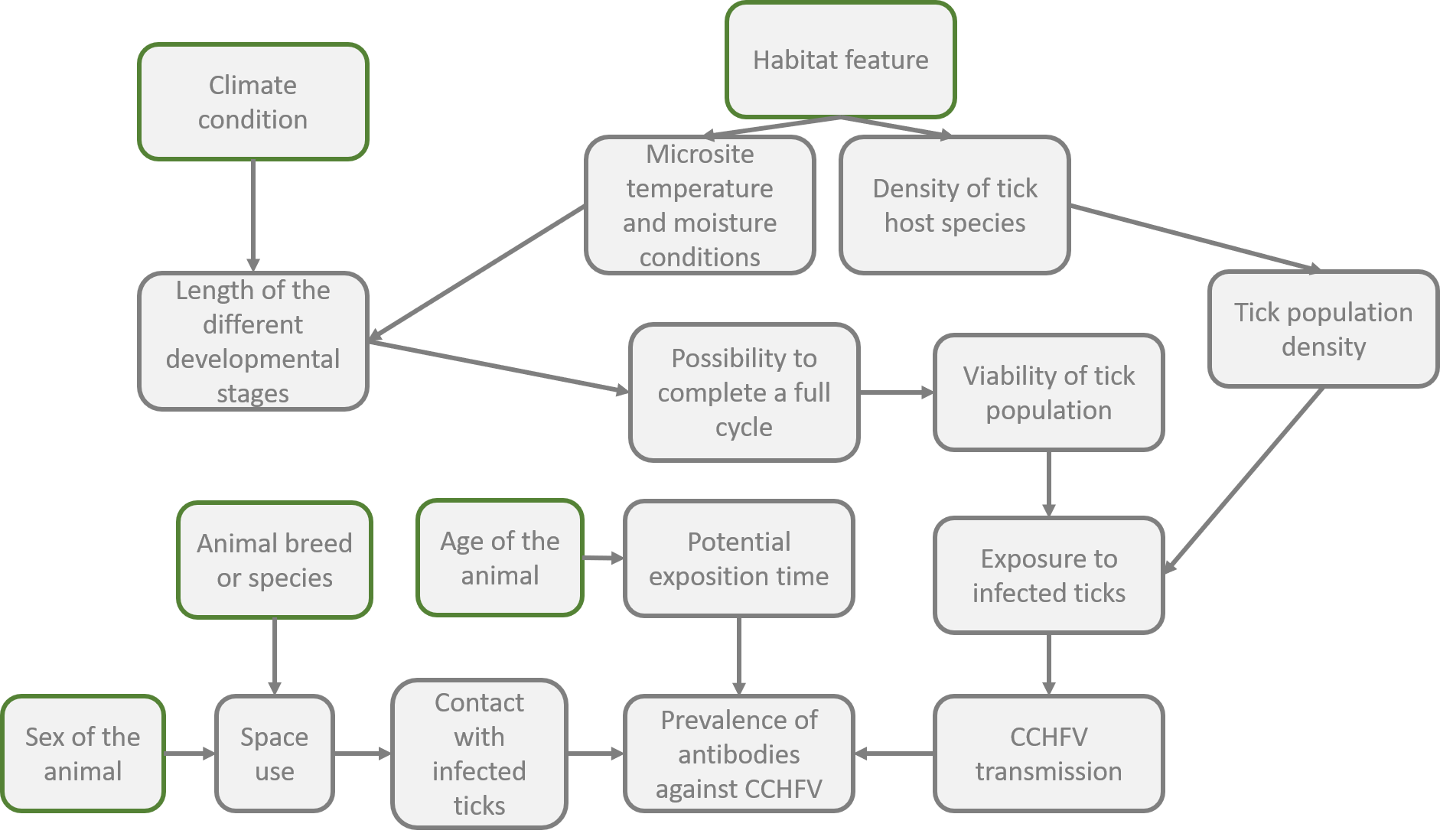

Supplement: S1 Fig — This conceptual framework was used to guide the selection of variables in multivariable hierarchical models and to reduce the risk of overadjustment or inclusion of collinear variables. (TIF) [file pone.0331875.s001.tif]
