# Supplementary material for: First detection of Crimean Congo Hemorrhagic Fever antibodies in cattle and wildlife of southern continental France: Investigation of explanatory factors
Source: PLoS One. 2025 Sep 24;20(9):e0331875. doi: 10.1371/journal.pone.0331875 (PMC12459774; doi:10.1371/journal.pone.0331875)
Supplement: S1 Table — (PDF) [file pone.0331875.s002.pdf]

Supplementary data 1 :

CODE	Dep	Species	OD	PPRNT
AM1	Alpes-maritimes	Cattle	69,00	160
AM10	Alpes-maritimes	Cattle	71,00	160
AM11	Alpes-maritimes	Cattle	66,00	<20
AM12	Alpes-maritimes	Cattle	75,00	160
AM13	Alpes-maritimes	Cattle	104,00	<20
AM14	Alpes-maritimes	Cattle	89,00	20
AM15	Alpes-maritimes	Cattle	40,00	20
AM16	Alpes-maritimes	Cattle	52,00	<20
AM17	Alpes-maritimes	Cattle	70,00	160
AM2	Alpes-maritimes	Cattle	74,00	<20
AM3	Alpes-maritimes	Cattle	81,00	40
AM4	Alpes-maritimes	Cattle	91,00	40
AM5	Alpes-maritimes	Cattle	43,00	<20
AM6	Alpes-maritimes	Cattle	50,00	80
AM7	Alpes-maritimes	Cattle	67,00	<20
AM8	Alpes-maritimes	Cattle	50,00	160
AM9	Alpes-maritimes	Cattle	123,00	160
BDR1	Bouches-du-Rhône	Cattle	31,00	<20
BDR2	Bouches-du-Rhône	Cattle	97,00	<20
BDR3	Bouches-du-Rhône	Cattle	45,00	<20
G1	Gard	Cattle	42,00	<20
G10	Gard	Cattle	34,00	<20
G11	Gard	Cattle	39,00	<20
G12	Gard	Cattle	55,00	<20
G13	Gard	Cattle	63,00	80
G3	Gard	Cattle	51,00	<20
G4	Gard	Cattle	54,00	<20
G5	Gard	Cattle	52,00	<20
G6	Gard	Cattle	71,00	<20
G9	Gard	Cattle	50,00	20
H10	Hérault	Cattle	211,00	80
H11	Hérault	Cattle	153,00	20
H12	Hérault	Cattle	63,00	40
H13	Hérault	Cattle	191,00	160
H14	Hérault	Cattle	158,00	40
H15	Hérault	Cattle	206,00	160
H16	Hérault	Cattle	206,00	160
H17	Hérault	Cattle	208,00	80
H18	Hérault	Cattle	201,00	160

H19	Hérault	Cattle	202,00	40
H2	Hérault	Cattle	116,00	80
H20	Hérault	Cattle	195,00	160
H22	Hérault	Cattle	130,00	<20
H3	Hérault	Cattle	198,00	160
H4	Hérault	Cattle	183,00	160
H5	Hérault	Cattle	178,00	160
H6	Hérault	Cattle	164,00	80
H7	Hérault	Cattle	164,00	20
H8	Hérault	Cattle	35,00	<20
H9	Hérault	Cattle	189,00	<20
PO1	Pyrénées-Orientales	Cattle	127,00	80
PO2	Pyrénées-Orientales	Cattle	112,00	160
PO3	Pyrénées-Orientales	Cattle	96,00	40
PO4	Pyrénées-Orientales	Cattle	107,00	80
PO5	Pyrénées-Orientales	Cattle	130,00	160
PO6	Pyrénées-Orientales	Cattle	152,00	160
PO7	Pyrénées-Orientales	Cattle	54,00	80
PO8	Pyrénées-Orientales	Cattle	140,00	40
PO9	Pyrénées-Orientales	Cattle	127,00	40
SR21	Hautes-Pyrénées	Red deer	65,33	40
SR22	Hautes-Pyrénées	Red deer	93,09	80
SR23	Hautes-Pyrénées	Red deer	112,85	160
SR24	Hautes-Pyrénées	Red deer	51,84	40
SR25	Hautes-Pyrénées	Red deer	37,26	80
SR26	Hautes-Pyrénées	Red deer	83,37	40
SR27	Hautes-Pyrénées	Roe deer	37,90	160
SR28	Hautes-Pyrénées	Roe deer	40,39	80
SR29	Hautes-Pyrénées	Roe deer	47,59	40
SR210	Hautes-Pyrénées	Red deer	39,79	<20
SR211	Hautes-Pyrénées	Roe deer	63,50	80
SR212	Hautes-Pyrénées	Roe deer	42,39	160
SR213	Hautes-Pyrénées	Red deer	66,87	160
SR214	Hautes-Pyrénées	Wildboar	52,95	40
SR215	Hautes-Pyrénées	Roe deer	108,38	<20
SR216	Hautes-Pyrénées	Wildboar	72,79	160
SR217	Hautes-Pyrénées	Roe deer	143,07	160
SR218	Hautes-Pyrénées	Wildboar	31,43	40
SR219	Hautes-Pyrénées	Red deer	101,11	160
SR220	Hautes-Pyrénées	Wildboar	87,77	80
SR221	Hautes-Pyrénées	Wildboar	54,71	40
SR222	Hautes-Pyrénées	Roe deer	76,37	20
SR223	Hautes-Pyrénées	Red deer	106,78	<20

SR224	Hautes-Pyrénées	Roe deer	106,87	160
SR225	Hautes-Pyrénées	Red deer	53,57	80
SR226	Hautes-Pyrénées	Wildboar	48,53	80
SR227	Hautes-Pyrénées	Red deer	97,39	80
SR228	Hautes-Pyrénées	Wildboar	74,90	160
SR229	Hautes-Pyrénées	Wildboar	124,46	160
SR230	Hautes-Pyrénées	Roe deer	51,76	160
SR231	Hautes-Pyrénées	Red deer	165,16	160
SR232	Hautes-Pyrénées	Red deer	92,60	160
SR233	Hautes-Pyrénées	Red deer	66,39	160
SR234	Hautes-Pyrénées	Red deer	92,23	160
SR235	Hautes-Pyrénées	Roe deer	133,52	160
SR236	Hautes-Pyrénées	Red deer	49,44	160
SR237	Hautes-Pyrénées	Roe deer	152,62	<20
SR238	Hautes-Pyrénées	Red deer	108,15	20
SR239	Hautes-Pyrénées	Roe deer	128,18	160
SR243	Lozère	Mouflon	39,45	80
