# Supplementary material for: First detection of Crimean Congo Hemorrhagic Fever antibodies in cattle and wildlife of southern continental France: Investigation of explanatory factors
Source: PLoS One. 2025 Sep 24;20(9):e0331875. doi: 10.1371/journal.pone.0331875 (PMC12459774; doi:10.1371/journal.pone.0331875)
Supplement: Attachment — Submitted filename: Response to Reviewers.pdf [file pone.0331875.s004.pdf]

Response to Reviewers – Manuscript PONE-D-25-20397

We thank the Academic Editor and the reviewers for their constructive and insightful comments. We have carefully revised the manuscript to address all points raised. Below, we provide detailed responses to each comment. All changes have been made in the revised manuscript and highlighted using tracked changes.

Reviewer #1

We thank the reviewer for their thoughtful overview and contextual framing of the current epidemiological situation of Crimean-Congo Hemorrhagic Fever Virus (CCHFV) in Europe. We fully agree that future integrated studies combining serological and virological approaches will be key to better mapping and understanding the dynamics of CCHFV transmission in Europe.

All the changes suggested in the document have been taken into account.

Comment : *As stated in the discussion section, lines : 367 -368"other tick species may also be effective." Therefore, in the introduction section, brief information can be given about tick diversity in regions where the disease has been detected, especially in Spain, and about possible vectors other than hyalomma spp. in France. It will be more informative for the readers.*

Response: We would like to thank the reviewer for his comment. We decided to add a sentence to be more informative. "Other tick species such as *Rhipicephalus bursa* and *Dermacentor marginatus* have occasionally been found carrying CCHFV in southwestern Europe, but their role in transmission remains uncertain. In France, despite the presence of multiple tick genera, only *H. marginatum* has been found positive to date".

Reviewer #2

We thank the reviewer for their constructive feedback and suggestions to improve the manuscript. Below, we provide point-by-point responses to each comment.

Comment: *1. Statistical modelling – given that many variables were offered for statistical modelling using hierarchical multivariable regression model, a formal approach for identifying orthogonal variables for inclusion must be used to improve the efficiency of the analysis. This will include using causal web models, directed acyclic curves etc.*

Response: Thank you for this suggestion. In response, we constructed a directed acyclic graph (DAG) to represent the hypothesized causal relationships between the exposure, explanatory variables, and CCHFV seropositivity in both livestock and wildlife. This DAG, based on literature and expert knowledge, was used to identify potential confounding structures and guide the variable selection process in the hierarchical multivariable models. The DAG is now provided as Supplementary Figure S1. In parallel, we performed correlation tests on the variables retained in our final models (after the dredge step and selection of the best-fitting model). These tests showed that the variables were not significantly correlated. In addition, mean comparison tests indicated that the group means were significantly different.

Comment: *2. The language should be reviewed to improve its presentation in consistent English.*

Response:
The entire manuscript has been thoroughly reviewed by a native English speaker and edited to ensure consistency, clarity, and correct grammar throughout.

Comment: *Line 43: CCHF has significant public health impacts in regions where the disease is endemic. So even if it does not expand its geographical range, the disease still has high public health significance. I suggest rewording this statement to reflect that fact.*

Response:
We agree and have reworded this sentence to reflect that CCHF remains a significant public health concern regardless of its geographic expansion.

Comment: *Line 69 and other places: replace "my study" with "our study"*

Response:
All instances of "my study" have been replaced with "our study" for consistency and appropriate authorship tone.

Comment: *Line 89 and 104: say "humans are infected" not "contaminated"*

Response:
We have replaced "contaminated" with "infected" in both instances (lines 89 and 104), which is the accurate term for human exposure to CCHFV.

Comment: *Line 91: Is WHO a reference?*

Response:
We have changed the reference in the manuscript.

Comment: *Line 143/148: delete "detecting" in the title since it is already implied in the sentence*

Response:
The term "detecting" has been removed from the respective sentences for conciseness and clarity (lines 143 and 148).

Comment: *Line 176 and 209: Start the line with text instead of a number (e.g., "(45)")*

Response:
We have revised the formatting to ensure that each sentence starts with text before the reference, as suggested.

Comment: *Line 529: use "spatial coverage"*

Response:
We have replaced the original term with "spatial coverage" in line 529.

Comment: *Line 530: put a full stop after "level"*

Response:
A full stop has been added after “level” (line 530).

Comment: *Line 587: use a different word instead of "demonizing"*

Response:
We have replaced “demonizing” with a more appropriate term (“stigmatizing”) to improve tone and clarity (line 587).

Comment: *Line 651: use "multivariable model" instead of "multivariate"*

Response:
We have corrected the term to “multivariable model” in line 651 to accurately reflect the modelling approach.

Journal Requirements

1. **Formatting**
   Response: The manuscript has been updated to match the PLOS ONE style and formatting requirements. File names have also been adjusted accordingly.
2. **LaTeX template**
   Response: We acknowledge the request to format our manuscript using the PLOS LaTeX template. However, due to technical constraints, we were unfortunately not able to implement this formatting for the current version of the revised submission. We hope the current formatting remains acceptable for evaluation purposes, and we remain available to reformat the manuscript using the template if required at a later stage.
3. **Financial disclosure update**
   Response: We have added the required sentence:

“The funders had no role in study design, data collection and analysis, decision to publish, or preparation of the manuscript.”
This has been added to both the cover letter and the manuscript.

4. **Figures 1–3 – Copyright compliance**
   Response: The maps used in Figures 1–3 were generated from publicly available data sources (e.g., Natural Earth, BD Forêt, CORINE Land Cover). We confirm that no copyrighted images (e.g., Google Maps) were used. All sources have been cited in the figure legends, and figures comply with the CC BY 4.0 license. Alternatively (if needed), reworked figures are provided to ensure compliance.
5. **Reference check**
   Response: We have reviewed all references and confirm that none are retracted. The reference list has been updated to ensure completeness and accuracy.
